# Elevated plasma cholesterol improves sepsis outcome by promoting hepatic metabolic reprogramming

**DOI:** 10.3389/fimmu.2026.1746724

**Published:** 2026-05-11

**Authors:** Qian Wang, Jianyao Xue, Ling Guo, Dan Hao, Misa Ito, Rianna Reese, Bin Huang, Congqing Wu, Xiang-An Li

**Affiliations:** 1 Saha Cardiovascular Research Center, University of Kentucky College of Medicine, Lexington, KY, United States; 2 Department of Pharmacology and Nutritional Sciences, University of Kentucky College of Medicine, Lexington, KY, United States; 3 University of Kentucky College of Medicine, , Highland Heights, KY, United States; 4 Division of Cancer Biostatistics; University of Kentucky College of Medicine, Lexington, KY, United States; 5 Department of Surgery, University of Kentucky College of Medicine, Lexington, KY, United States; 6 Department of Microbiology, Immunology and Molecular Genetics, University of Kentucky College of Medicine, Lexington, KY, United States; 7 Lexington VA Healthcare System, Lexington, KY, United States; 8 Department of Physiology, University of Kentucky College of Medicine, Lexington, KY, United States

**Keywords:** cholesterol, high cholesterol diet (HCD), metabolic reprogramming, oxidative phosphorylation, sepsis

## Abstract

**Background:**

Sepsis is a life-threatening condition with high mortality and limited therapeutic options. This study investigated the association between plasma cholesterol levels and sepsis survival and explored the mechanisms by which elevated cholesterol confers protection.

**Methods:**

We analyzed 2,787 sepsis patients from the MIMIC-IV database, comparing cholesterol levels between 28-day survivors and non-survivors and assessing mortality risk using multivariable Cox regression. To test causality, C57BL/6J mice were fed either a high-cholesterol diet (HCD) or a regular diet (RD) before cecal ligation and puncture (CLP).

**Results:**

Survivors had significantly higher cholesterol than non-survivors (median 135 vs. 126 mg/dL; p < 0.001). High cholesterol (≥133 mg/dL) was independently associated with reduced 28-day mortality (adjusted HR = 0.80; 95% CI: 0.67–0.95; p = 0.012). In mice, HCD elevated plasma cholesterol and improved survival (52.5% to 90%), through a mechanism that is not primarily explained by broad immune activation. Hepatic transcriptomics revealed metabolic reprogramming, including enhanced oxidative phosphorylation and antioxidant pathways, with suppression of endoplasmic reticulum proteostasis. Inhibition of mitochondrial respiration abolished the survival benefit.

**Conclusions:**

Elevated plasma cholesterol is associated with improved sepsis outcomes, likely through promoting hepatic metabolic reprogramming. Targeting hepatic bioenergetics is a potential therapeutic approach.

## Introduction

Sepsis is a life-threatening condition arising from a dysregulated host response to infection, leading to organ dysfunction and death (1, 2). Despite extensive efforts to target the inflammatory cascade—once considered the primary driver of sepsis—therapeutic interventions have yielded only modest survival benefits (3). This underscores the urgent need to deepen our mechanistic understanding of sepsis and to identify novel therapeutic strategies.

Hypocholesterolemia is frequently observed in septic patients (4–7), and existing evidence suggests an inverse relationship between plasma cholesterol levels and survival in sepsis (8–11). However, these early studies were limited by small sample sizes, typically ranging from dozens to a few hundred patients, which restricts the ability to account for confounding factors. A recent meta-analysis involving 2,542 critically ill patients adjusted only for sex and age (11), leaving significant gaps in understanding. Therefore, larger and more comprehensive clinical studies are needed to clarify these associations.

Cholesterol, a vital component of cellular membranes, plays critical roles in maintaining membrane integrity, modulating immune responses, and regulating cellular metabolism (12). Beyond its structural role, cholesterol actively participates in host defense (13). Lipoproteins can bind and neutralize bacterial endotoxins, modulate lipid raft–dependent innate immune signaling, and influence cellular stress responses (14). Emerging evidence suggests that cholesterol availability also affects mitochondrial function (15), redox homeostasis, and cellular energy metabolism—processes that are profoundly disrupted during sepsis. Nevertheless, the biological mechanisms linking cholesterol homeostasis to sepsis severity and survival remain incompletely understood.

Importantly, sepsis is increasingly recognized as a syndrome of metabolic failure rather than solely an immunological disorder. Mitochondrial dysfunction, impaired oxidative phosphorylation, and excessive oxidative stress are central drivers of organ failure and mortality (16). Yet how systemic lipid availability interfaces with these bioenergetic pathways has received limited attention. Most prior work has focused on immune modulation, leaving a critical gap in understanding whether cholesterol contributes to metabolic adaptation and stress tolerance during severe infection. Elucidating the underlying mechanisms may provide insights into the pathogenesis of sepsis and reveal novel therapeutic targets.

To address these gaps, we integrated clinical data analysis from the MIMIC-IV database with mechanistic investigations in a murine model of sepsis. Our findings reveal that lower plasma cholesterol levels are significantly associated with increased mortality, and that mice fed a high-cholesterol diet (HCD) for three days are markedly protected from cecal ligation and puncture (CLP)-induced sepsis. Mechanistic studies further demonstrate that HCD feeding promotes metabolic reprogramming characterized by enhanced oxidative phosphorylation, glutathione-mediated antioxidant defenses, and suppression of endoplasmic reticulum proteostasis. These insights suggest that cholesterol may act as a critical modulator of host resilience during severe infection, offering new avenues for therapeutic intervention through metabolic pathway targeting in sepsis.

## Experimental procedures

### Materials and methods

Materials are listed in the Supplemental Major Resources Table.

### Retrospective study

The retrospective study utilized the Medical Information Mart for Intensive Care IV (MIMIC-IV) version 3.1, a publicly accessible database containing de-identified health-related data from patients admitted to intensive care units at the Beth Israel Deaconess Medical Center between 2008 and 2022. We loaded the MIMIC-IV dataset in the original CSV file format into DuckDB v1.3.2. We adopted many SQL code from MIMIC GitHub (https://github.com/MIT-LCP/mimic-code) to generate derived tables for data analysis in R v4.4.3. For the analysis, the first total cholesterol measurement within the first 24 hours of ICU admission was used. If ICU data were unavailable, the first total cholesterol measurement obtained during the same hospital stay was used instead. All code related to data analysis in the study will be publicly available upon publication.

### Patient inclusion

In the MIMIC-IV database (v3.1), a total of 94458 unique ICU stays were identified. Patients were excluded based on the following criteria (1): not admitted to the ICU for the first time (n=29092) (2); underwent cardiothoracic surgery (n=11642) (3); not diagnosed with sepsis according to Sepsis-3 criteria (n=54579); and (4) total cholesterol not available (n=18205). The final study cohort included 2787 patients. A flowchart outlining the data extraction and patient selection process is provided in **Supplementary Figure 1**.

### Mice and diet

C57BL/6J (B6) mice were purchased from Jackson Laboratory. Both male and female mice were used in experiments. B6 mice were randomized to feed on a high cholesterol diet (HCD) (TD. 88051, 7.5% cocoa butter, 15.8% fat, 1.25% cholesterol, 0.5% sodium cholate) or a regular-control diet (RD) (2918 Teklad Irradiated Global 18% Protein Rodent Diet, 4.5% fat, 0.022% cholesterol). Diets were administered for 3 days prior to procedures and maintained postoperatively. The HCD contains sodium cholate, which facilitates intestinal absorption of dietary cholesterol while limiting its fecal elimination and is therefore commonly used in animal models to induce hypercholesterolemia and cholesterol-driven pathophysiological processes. To evaluate the independent effect of sodium cholate on circulating cholesterol levels, B6 mice were fed a standard chow diet supplemented with 0.5% sodium cholate for 7 days. When euthanizing mice, the mice were anesthetized. Animal experiments were approved by the Animal Care and Use Committee of the University of Kentucky.

### Rotenone treatment

Rotenone (Sigma-Aldrich, R8875) was dissolved in ethanol (90 mg/100 ml). This solution was evenly sprayed onto 200g of HCD and allowed to dry thoroughly for one week prior to feeding. The dosing strategy was guided by prior studies (17), assuming each mouse consumes 2g of diet/day.

### CLP-induced sepsis model

CLP was performed on about 3-month-old mice as previously described (18). Mice were anesthetized using an isoflurane vaporizer machine delivering isoflurane in 100% oxygen. Anesthesia was induced by placing each mouse under a nose cone delivering 5% isoflurane until loss of the righting reflex and absence of a pedal withdrawal response. After induction, the mouse was positioned on a surgical platform, and anesthesia was maintained at 3.5% isoflurane via the same nose−cone setup for the duration of the procedure. Throughout surgery, respiratory rate and pedal reflexes were monitored to ensure adequate anesthetic depth. The cecum was exteriorized, ligated with a 4−0 silk ligature at the midpoint, and punctured twice with a 23G needle to induce polymicrobial sepsis. Mice were monitored for survival for 7 days following CLP. For mechanistic studies, mice were anesthetized as described above, and peritoneal fluids, blood, liver, and other organs were collected at the indicated time points.

### Biochemical assays

Blood was collected from the abdominal aorta at 0, 4, 20, and 44 hours post-CLP. Serum was separated for biochemical assays. The cholesterol kit was from Wako. Serum cytokines were analyzed by Eve Technologies using Mouse Cytokine Array/Chemokine Array 32-Plex (MD32). Corticosterone was measured with an ENZO Life Science kit. Blood glucose levels were measured with a glucose measurement kit (Glucose Meter Kit, Contour) or glucose measurement strips (Glucose test strips, Contour).

### Coomassie blue staining of serum albumin

Serum samples collected 4 hours after CLP were used for albumin detection. Each sample was diluted 1:40 in distilled water, and 10 µL of the diluted serum was loaded per well onto a 10% SDS-PAGE. Following electrophoresis, gels were stained with Coomassie Brilliant Blue to visualize albumin bands. After staining, the gels were destained until a clear background was achieved. Albumin bands were visualized and quantified by densitometric analysis using ImageJ software, with band intensity normalized to the same total protein input across all lanes for comparison between groups.

### Hematoxylin and eosin

Liver tissues were harvested after 3 days of HCD feeding and immediately fixed in 10% formalin. Fixed tissues were dehydrated through graded ethanol, cleared in xylene, and embedded in paraffin. Paraffin-embedded liver samples were sectioned at 5 µm thickness and mounted on glass slides. Sections were deparaffinized, rehydrated, and stained with hematoxylin and eosin. Stained sections were dehydrated, cleared, and coverslipped for histological examination. Images were acquired using a light microscope.

### Bacterial load

Blood, peritoneal fluid, spleen, and liver from CLP mice were homogenized and diluted (20, 200, and 2000 times with dH2O), and 100 μL of the dilution was plated on an LB-Agar plate and incubated at 37°C for 24h, and then the number of clones was counted.

### Flow cytometry analysis of leukocyte recruitment and splenic immunocytes

Leukocyte recruitment to the peritoneal cavity and immunocyte profiling in the spleen were assessed by flow cytometry. Mice were sacrificed at 4 h, 20 h, and 44 h post-CLP. To collect peritoneal cells, 5 mL of PBS was injected into the peritoneal cavity, and the peritoneal fluid was harvested. Total cell numbers were counted, and 1×10^6^ cells were used for staining. Splenic immunocytes were analyzed from HCD and RD mice before CLP and at 20 h post-CLP. After red blood cell lysis, 1×10^6^ splenocytes were prepared for flow cytometry. In both protocols, cells were blocked with CD16/32 antibody to eliminate nonspecific Fc receptor binding. Peritoneal cells were stained with Ly-6C-FITC, Ly-6G-PE, CD11b-PerCP-5.5, and CD45-APC. After gating on singlets and viable cells, leukocytes were identified as CD45^+^ cells, and myeloid cells were further gated as CD45^+^CD11b^+^ cells. Within this population, peritoneal neutrophils (PMNs) were defined as CD45^+^CD11b^+^Ly6G^+^Ly6C^hi^ cells, and inflammatory monocytes (IMs) were defined as CD45^+^CD11b^+^Ly6G-Ly6C^hi^ cells based on Ly6G and Ly6C expression (19). Splenocytes were stained with CD45-APC, B220-FITC, CD3-PE/Cy7, and CD11b-PerCP/Cy5.5. B cells (B220^+^), T cells (CD3^+^), and myeloid cells (CD11b^+^) were identified within the CD45^+^ population. All staining was performed for 30 minutes at 4°C, followed by washing with FACS buffer. Samples were analyzed using a BD LSRII Flow Cytometer.

### RNA-seq analysis

The liver was collected from mice after 3-day HCD or RD. Total RNA was isolated using the RNeasy Kit. Sequencing and data analysis were performed by Novogene. Genes with an adjusted p-value (FDR < 0.05) and an absolute log_2_ fold change > 0 were considered differentially expressed genes (DEGs). Pathway enrichment analysis of DEGs was performed using Kyoto Encyclopedia of Genes. Gene set enrichment analysis (GSEA) was conducted using all detected genes to assess enrichment of Gene Ontology (GO) bile acid signaling pathway.

### Western blot analysis of OXPHOS proteins

Liver tissues were collected 20 h after CLP. Approximately 35 mg of liver tissue per sample was homogenized in MBST/OG buffer supplemented with protease inhibitor. Total protein concentrations were determined using a BCA assay. Equal amounts of protein were resolved by 12.5% SDS–PAGE gels and transferred onto PVDF membranes. Membranes were incubated with primary antibodies against mitochondrial oxidative phosphorylation (OXPHOS) complexes I–V (45−8099, Invitrogen), followed by anti−mouse secondary antibodies. Band intensities were quantified using ImageJ software.

### Statistical analysis

Survival data were analyzed using the Log-Rank test and Kaplan-Meier plots. Comparisons between two groups were performed using a two-tailed Student’s t-test, while comparisons among multiple groups were analyzed using two-way ANOVA. Statistical analyses were conducted using GraphPad software. Data are presented as mean ± SEM, and differences were considered statistically significant at p < 0.05.

## Results

### Low plasma cholesterol concentration correlates with a poor prognosis in septic patients

**Figure 1:**
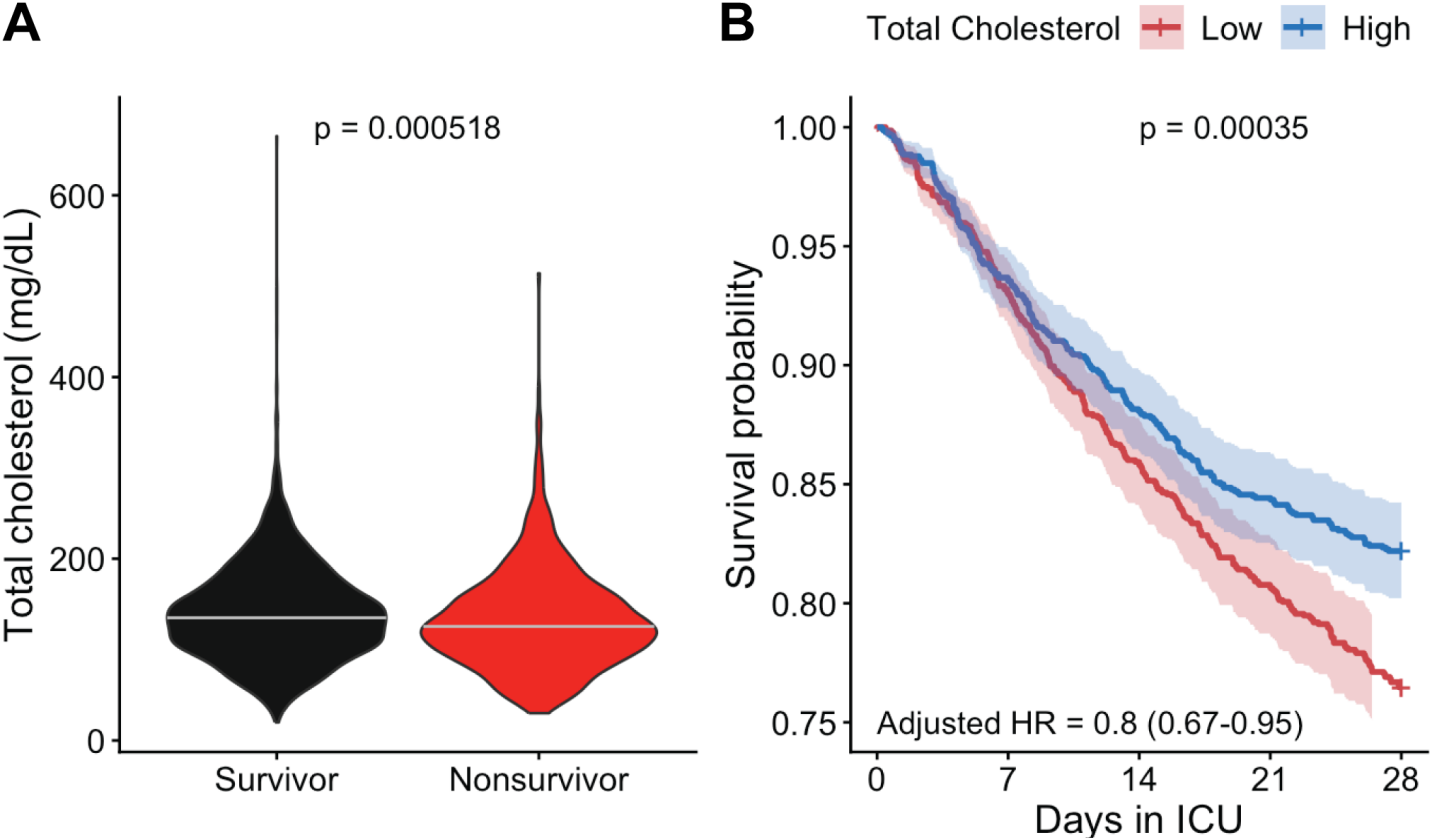
Low plasma cholesterol concentration correlates with a poor prognosis in septic patients. **(A)** Distribution of total cholesterol levels by 28-day mortality status. Survivors had significantly higher median total cholesterol levels (within 24 hours of ICU admission), p = 0.000518, Wilcoxon rank-sum test. **(B)** Kaplan-Meier survival curves comparing patients with high versus low total cholesterol levels (dichotomized at the median of 133 mg/dL) showed significantly worse 28-day survival in the low-cholesterol group (log-rank p = 0.00035). In the Cox proportional hazards model, adjusted for age, gender, race, SOFA score, and Charlson comorbidity index, higher cholesterol was associated with reduced mortality (adjusted HR = 0.8, 95% CI: 0.67-0.95, p = 0.012).

To better understand the relationship between plasma cholesterol levels and sepsis outcomes, we analyzed data from 2787 sepsis patients (sepsis-3 criteria (1)) with total cholesterol measurements in the MIMIC-IV v3.1 database (20). Baseline characteristics of survivors and non-survivors are summarized in **Supplementary Table 1**. Non-survivors were older (70.6 vs. 64.9 years), had higher SOFA scores (6.1 vs. 5.4), and higher Charlson comorbidity indices (6.8 vs. 5.5). Survivors had significantly higher median total cholesterol levels compared with non-survivors (135 mg/dL vs 126 mg/dL, p < 0.001), demonstrating a significant inverse association between total cholesterol levels and 28-day mortality (**Figure 1A**). Patients with higher cholesterol levels showed improved survival outcomes (log-rank, p = 0.00035), with an adjusted (age, gender, race, SOFA score within the first 24 hours, and Charlson comorbidity index) hazard ratio of 0.80 (95% CI: 0.67-0.95, p = 0.012) for high versus low cholesterol groups (**Figure 1B**). These adjustments account for major baseline differences in disease severity and comorbidity burden between groups. This finding supports the notion that low plasma cholesterol concentrations are a risk factor for sepsis death.

### High-cholesterol diet protects against polymicrobial sepsis

**Figure 2:**
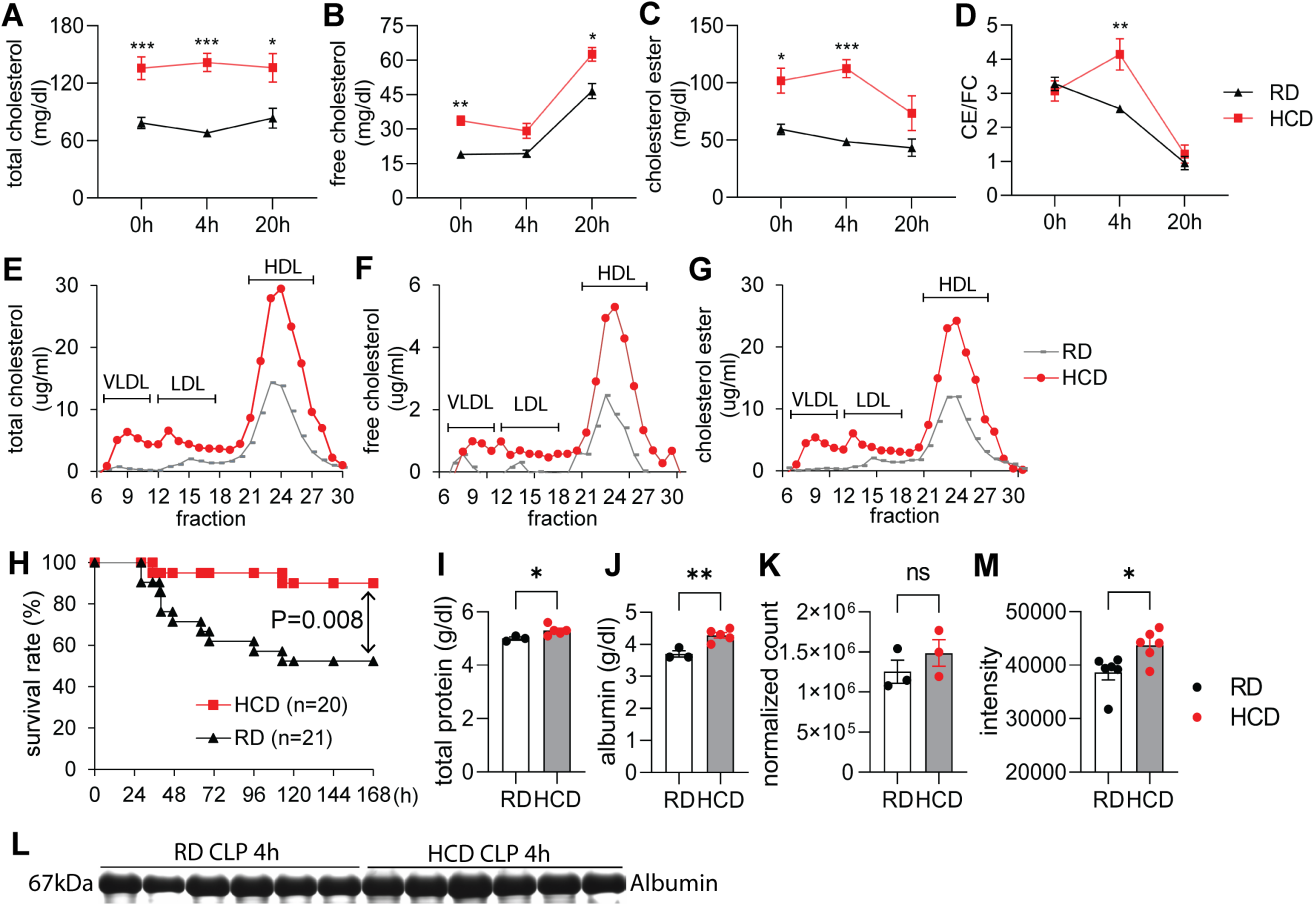
High-cholesterol diet protects against polymicrobial sepsis. C57BL/6J mice were fed a regular diet (RD) or high-cholesterol diet (HCD) for 3 days, followed by induction of sepsis via cecal ligation and puncture (CLP). **(A–D)** Serum concentration of **(A)** total cholesterol, **(B)** free cholesterol (FC), **(C)** cholesteryl ester (CE), and **(D)** CE/FC ratio at 0, 4, and 20 hours post-CLP. RD, n = 8–20; HCD, n = 6–10 per time point. **(E–G)** Fast protein liquid chromatography (FPLC) profiles of serum **(E)** total cholesterol, **(F)** free cholesterol (FC), and **(G)** cholesterol ester (CE) levels after 3 days of feeding (n = 2 per group, representative FPLC profiling). **(H)** Survival analysis post-CLP, assessed by the log-rank test. **(I–K)** Serum total protein **(I)** and albumin **(J)** levels, and RNA sequencing analysis of Albumin gene expression in the liver **(K)** following a 3-day diet. **(L, M)** Serum was collected 4 hours after cecal ligation and puncture (CLP), and an equal volume of serum was applied to SDS-PAGE. The gel was stained with Coomassie blue **(L)**, and serum albumin was quantified **(M)**. Data are presented as mean ± SEM; Statistical significance was determined by two-way ANOVA **(A–D)**, Log-Rank test **(H)**, or t-test **(I–M)**. *p < 0.05, **p < 0.01, ***p < 0.001 for HCD vs. RD comparisons; ns, not significant.

To test this hypothesis, we fed C57BL/6J mice with either a high-cholesterol diet (HCD) or a regular diet (RD) for three days. HCD feeding significantly increased plasma cholesterol levels from 78 to 136 mg/dl without altering the cholesterol ester-to-free cholesterol (CE/FC) ratio (**Figures 2A–D**). Lipoprotein profiling showed increased cholesterol across all lipoprotein fractions in HCD-fed mice (**Figures 2E–G**). Following cecal ligation and puncture (CLP), HCD-fed mice maintained substantially higher plasma cholesterol concentrations throughout the septic course compared to RD-fed mice (**Figures 2A–C**). Notably, the CE/FC ratio was increased in the HCD group at 4 hours post-CLP (**Figure 2D**). Because sex can influence cholesterol metabolism and immune responses (21), we analyzed plasma cholesterol levels separately in male and female mice. Short-term high-cholesterol diet feeding resulted in an increase in total cholesterol in both sexes at baseline and following CLP (**Supplementary Figures 2A, B**). We also measured cholesterol levels in mice fed a regular diet containing cholate. Consistent with the previous report (22, 23), cholate alone did not significantly increase cholesterol levels in a regular diet (**Supplementary Figure 2C**). Importantly, HCD feeding significantly increased survival from 52.5% to 90% (**Figure 2H**), which is consistent with our clinical observations.

To explore protective mechanisms, we assessed organ injury markers—plasma alanine aminotransferase (ALT) for liver injury and blood urea nitrogen (BUN) for kidney damage—and found no significant differences between HCD- and RD-fed mice at 4, 20, and 44 hours post-CLP (**Supplementary Figures 3A, B**). Consistent with these biochemical findings, histological analysis of liver sections revealed no overt differences in hepatic architecture between dietary groups (**Supplementary Figure 2C**), indicating that HCD feeding does not exacerbate liver injury during sepsis. Given the metabolic nature of the dietary intervention, we next evaluated serum glucose. While HCD-fed mice exhibited modestly higher glucose levels under basal conditions, RD-fed mice showed significantly higher glucose concentrations at 4 hours post-CLP (**Supplementary Figure 2D**), suggesting differential metabolic responses to early septic stress rather than sustained hyperglycemia induced by HCD feeding. We next evaluated the inflammatory response by measuring 32 serum cytokines and nitric oxide metabolites (NOx). Only a few cytokines differed between groups: IL-1β, KC, and MIP-2 were higher in RD-fed mice at 4 h after CLP; IL-9 at 20 h; and G-CSF at 44 h (P < 0.05; **Supplementary Figure 3E**; **Table 2**). There were no differences in NOx levels (**Supplementary Figure 3F**). Flow cytometry analysis revealed minor differences in leukocyte recruitment to the peritoneal cavity (**Supplementary Figure 4**). In the spleen, HCD feeding was associated with an increase in the percentage of macrophages (P < 0.05), as well as increased absolute numbers of B cells (P < 0.05), macrophages (P < 0.001), and T cells (P < 0.001) (**Supplementary Figure 5**). To determine whether these modest changes affected bacterial clearance, we quantified bacterial loads in blood, peritoneal fluid, and organs, and observed no significant differences (**Supplementary Figure 6**). These findings suggest that elevated plasma cholesterol–associated protection is unlikely to be primarily mediated by broad inflammatory changes or enhanced bacterial clearance.

Because sepsis induces adrenal glucocorticoid (GC) synthesis, which uses cholesterol as a substrate, we measured corticosterone levels. Both groups exhibited a marked rise at 4 hours post-CLP, with no significant differences between HCD and RD mice (**Supplementary Figure 3G**).

**Figure 3:**
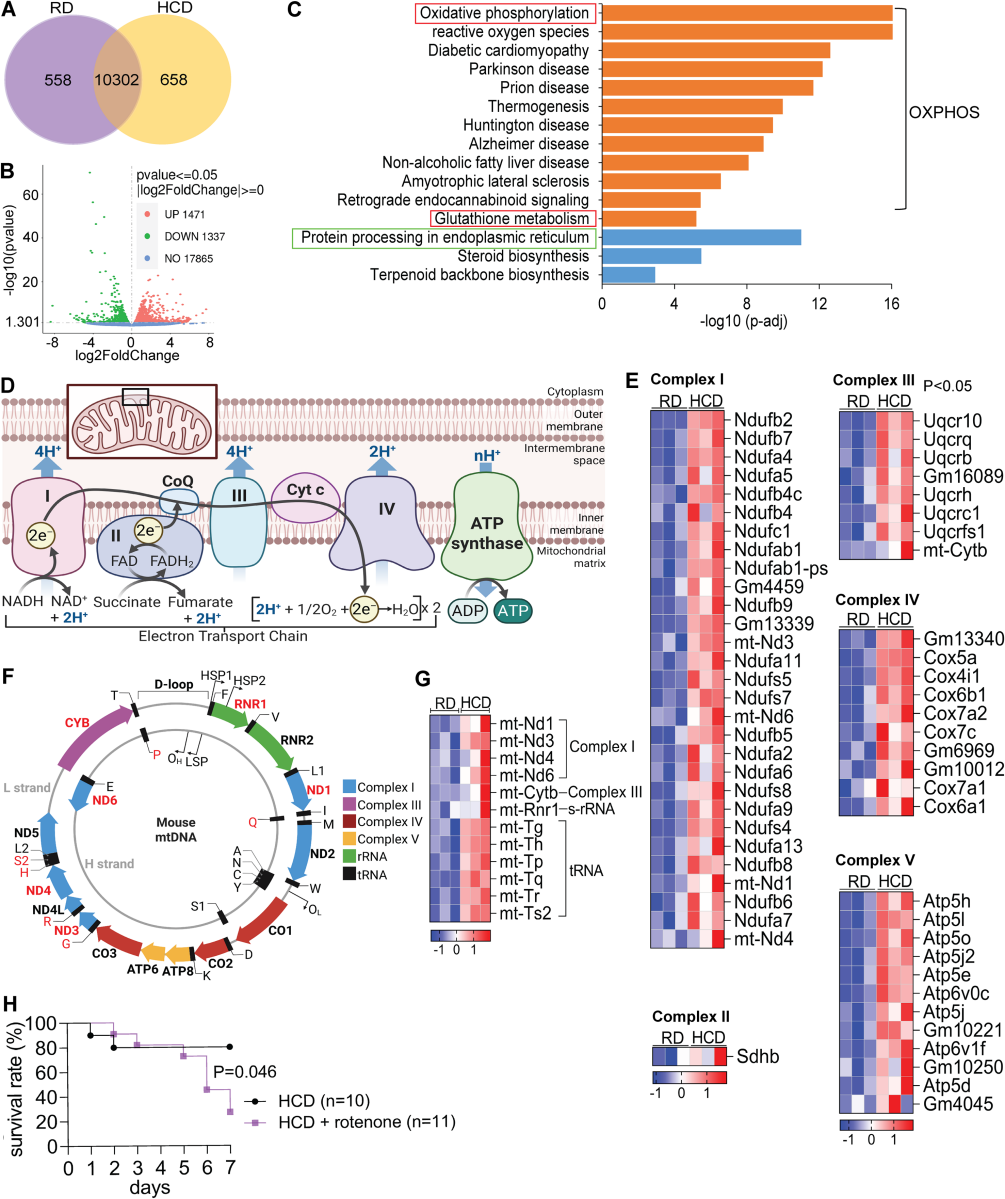
High-cholesterol diet induces metabolic reprogramming in the liver that protects against sepsis. C57BL/6J mice were fed a regular diet (RD) or high-cholesterol diet (HCD) for 3 days, followed by liver RNA sequencing (n = 3 per group). **(A)** Venn diagram showing the distribution of overlapping and unique differentially expressed genes (DEGs) between the HCD and RD groups. **(B)** RNA-seq analysis identifies altered genes in HCD-fed mice. **(C)** Top 12 upregulated pathways identified by KEGG (orange bar) and downregulated pathways (blue bar). **(D)** Schematic of mitochondrial respiratory chain complexes I–V. **(E)** Heatmap of upregulated differentially expressed genes (DEGs) in mitochondrial respiratory chain complexes I–V (p < 0.05). **(F)** Schematic of mitochondrial-encoded genes. **(G)** Heatmap of upregulated DEGs in mitochondrial DNA encoded genes. **(H)** Survival analysis of wild-type mice fed RD or HCD and treated with or without rotenone (a mitochondrial complex I inhibitor) following CLP-induced sepsis. Rotenone was administered by switching mice from a HCD to a rotenone-containing HCD for one night prior to CLP. Survival was assessed using the Log-Rank test.

Blood chemistry analysis revealed significantly higher plasma total protein and albumin in HCD-fed mice (**Figures 2I, J**) without a significant change in Albumin gene expression in the liver (**Figure 3K**). Four hours post-CLP, the HCD-fed mice still had higher albumin levels compared to RD-fed mice (**Figures 2L, M**). These suggest potential metabolic alterations.

### High-cholesterol diet induces metabolic reprogramming in the liver that protects against sepsis

To further explore the mechanisms, we performed RNA-seq analysis of liver tissue. As shown in **Figure 3A**, we identified 558 and 658 unique genes expressed in the RD and HCD groups, respectively. Differential expression analysis revealed 1,471 upregulated and 1,337 downregulated genes in HCD-fed mice compared to RD controls (**Figure 3B**).

KEGG pathway enrichment analysis highlighted oxidative phosphorylation (OXPHOS) as the most significantly upregulated pathway (adj. P = 5.01 × 10^-17^; **Figure 3C**). This was accompanied by coordinated transcriptional activation of both nuclear- and mitochondrial-encoded genes across respiratory chain complexes I–V (**Figures 3D–G**). Of the 65 genes encoding these complexes, 60 were significantly upregulated (**Figure 3E**), including mitochondrial DNA-encoded genes such as Nd1, Nd3, Nd4, Nd6, and Cytb (**Figures 3F, G**). Interestingly, the top 11 KEGG pathways —including diabetic cardiomyopathy, prion disease, thermogenesis, Parkinson’s disease, and Alzheimer’s disease—shared substantial overlap in upregulated genes with the OXPHOS pathway (**Supplementary Figure 7A**). This suggests that their enrichment may reflect shared mitochondrial gene activation rather than distinct disease-specific processes. Taken together, these findings indicate that HCD induces a pronounced mitochondrial metabolic shift in the liver, characterized by enhanced OXPHOS activity.

**Figure 4:**
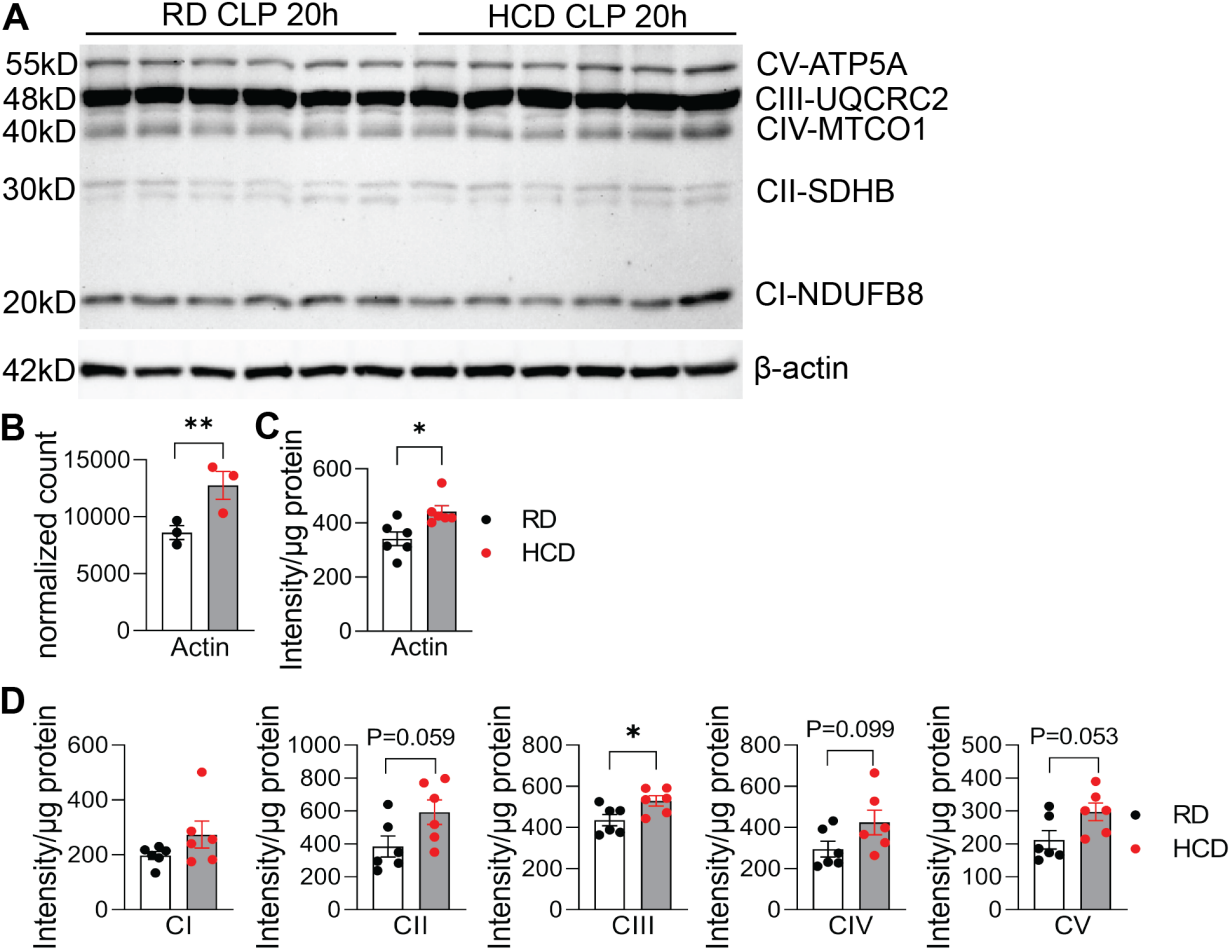
High-cholesterol diet enhances hepatic OXPHOS protein expression following CLP. Liver samples from HCD- and regular diet (RD)-fed mice were collected at 20 hours post-CLP and analyzed by Western blot. **(A)** Representative Western blots showing expression of mitochondrial oxidative phosphorylation (OXPHOS) complexes I–V (CI–CV). **(B)** RNA normalized counts of Actin in liver tissue after 3 days of HCD feeding prior to CLP. **(C)** Corresponding Actin protein-level quantification at 20 hours post-CLP. **(D)** Quantification of OXPHOS complex protein levels, normalized to total protein loading. Data are presented as mean ± SEM. *p < 0.05, **p < 0.01 by Student’s t‑test.

To complement the RNA-seq findings, we performed protein-level validation of mitochondrial OXPHOS components by Western blot analysis (**Figure 4**). A total protein normalization strategy was used, as β-Actin was upregulated in the HCD group, making it unsuitable as a loading control (**Figures 4B, C**). After protein normalization, Complex III (CIII) protein levels were significantly increased in HCD-fed mice, with trends toward increased expression of Complex II (CII, p = 0.059) and Complex V (CV, p = 0.053) (**Figures 4A, D****).** These protein-level changes became evident at 20 hours post-CLP, indicating a temporal delay between transcriptional and protein-level responses.

**Figure 5:**
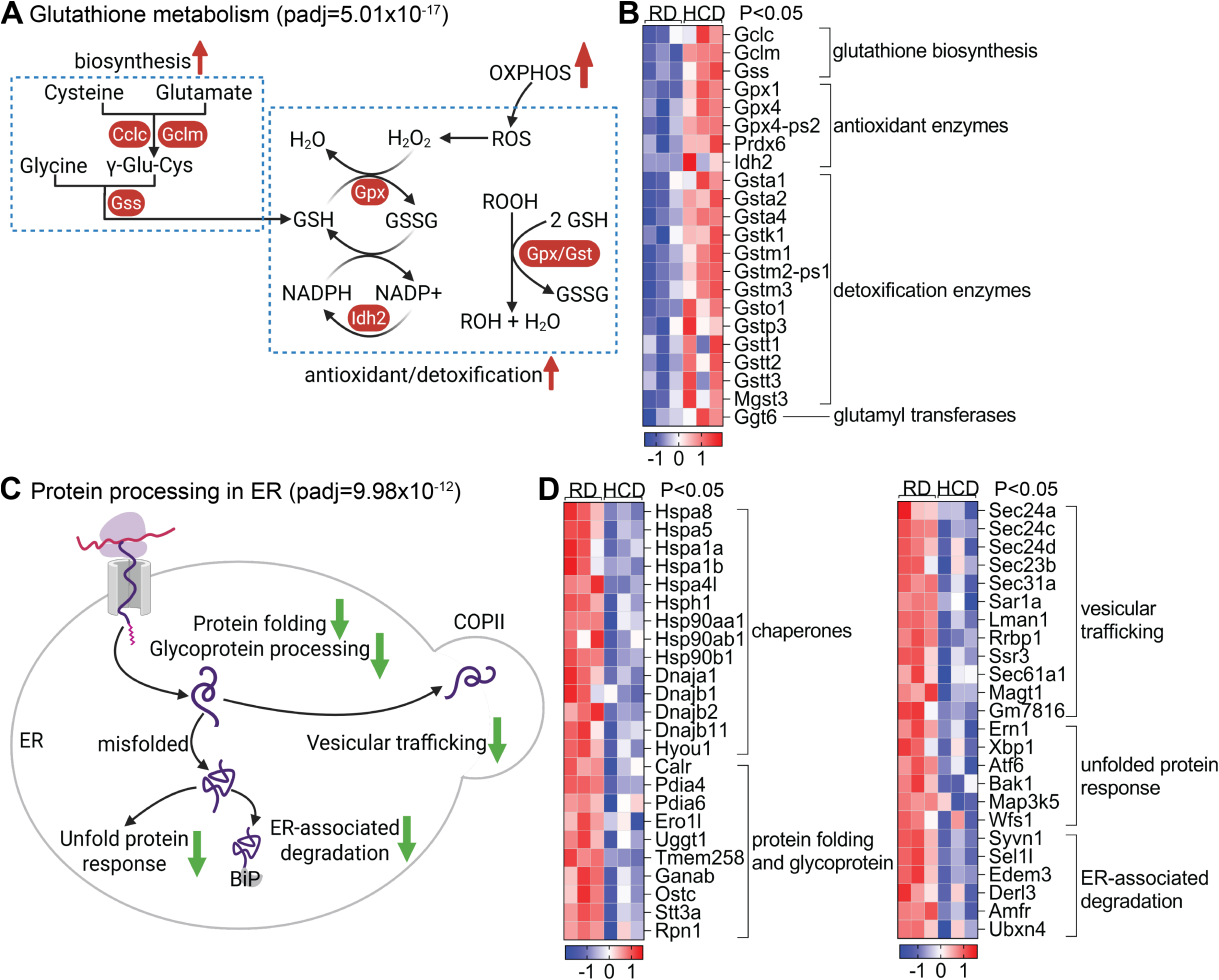
High-cholesterol diet promotes metabolic reprogramming - upregulating glutathione metabolism and downregulating protein processing in the endoplasmic reticulum (ER). RNA sequencing analysis was conducted on liver tissues from mice fed a high-cholesterol diet (HCD) or a regular diet (RD). **(A)** KEGG pathway enrichment analysis revealed significant upregulation of the glutathione metabolic pathway. Glutathione (GSH) biosynthesis: GSH, a tripeptide of glutamate, cysteine, and glycine, is synthesized in two ATP-dependent steps: γ-glutamylcysteine ligase (comprising catalytic GCLC and modifier GCLM subunits) forms γ-glutamylcysteine, and glutathione synthetase (GSS) subsequently adds glycine to complete GSH, with the first step serving as the rate-limiting reaction. Antioxidant/detoxification: Enhanced mitochondrial oxidative phosphorylation (OXPHOS) elevates reactive oxygen species (ROS) production, with superoxide rapidly converted to hydrogen peroxide (H_2_O_2_). Glutathione peroxidases (Gpx) detoxify H_2_O_2_ and organic hydroperoxides (ROOH) by reducing them with glutathione (GSH), generating H_2_O and oxidized glutathione (GSSG). Glutathione reductase then recycles GSSG back to GSH using NADPH, sustaining the GSH/GSSG redox cycle that preserves cellular redox balance and mitochondrial function under high bioenergetic demand. **(B)** Heatmap showing upregulated differentially expressed genes (DEGs) in glutathione biosynthesis and in antioxidation (p < 0.05). **(C)** KEGG pathway enrichment analysis revealed significant downregulation of protein processing in the ER pathway. Protein processing in the endoplasmic reticulum (ER) involves coordinated pathways that ensure proper protein maturation and quality control. Newly synthesized polypeptides undergo folding and glycoprotein processing, while misfolded proteins are recognized and targeted for ER-associated degradation (ERAD). Vesicular trafficking facilitates the export of correctly folded proteins, whereas accumulation of unfolded or misfolded proteins activates the unfolded protein response (UPR), which restores ER homeostasis by enhancing folding capacity, reducing protein load, and promoting degradation pathways. **(D)** Heatmap showing downregulated DEGs in the ER protein processing pathway. (p < 0.05).

Activation of OXPHOS pathways can increase oxidative stress, prompting us to examine the corresponding antioxidant responses. KEGG pathway analysis identified glutathione metabolism as the most significantly upregulated pathway after OXPHOS (adj. P = 2.32 × 10^-7^; **Figure 3C**). A broad array of antioxidant defense genes was upregulated (**Figures 5A, B**). Glutathione metabolism is closely linked to oxidative phosphorylation by maintaining mitochondrial redox homeostasis during aerobic energy production. Because electron transport inevitably generates reactive oxygen species, an efficient glutathione system is required to protect mitochondrial proteins, lipids, and DNA from oxidative damage. Reduced glutathione acts as a central redox buffer, supporting glutathione peroxidase–mediated peroxide detoxification and preserving electron transport chain function. Enhanced glutathione availability or recycling therefore limits oxidative stress, sustains electron flux and ATP production, and preserves overall mitochondrial bioenergetic capacity (24). Key enzymes involved in glutathione biosynthesis and recycling were upregulated, including Gclc and Gclm (which form the rate-limiting enzyme glutamate-cysteine ligase), and Gss (glutathione synthetase). Glutathione peroxidases (Gpx1, Gpx4, Gpx4-ps2) and Prdx6 were also elevated, indicating enhanced enzymatic reduction of hydrogen peroxide and lipid peroxides. Multiple glutathione S-transferases (Gstm2-ps1, Gsta2, Gsta4, Gstm1, Mgst3, Gsto1, Gstt2, Gstm3, Gstt3, Gstp3, Gsta1, Gstt1, Gstk1), catalyze the conjugation of glutathione to electrophilic compounds, facilitating their detoxification. In addition, elevated expression of Idh2 and Ggt6 further supports increased NADPH production and glutathione turnover, essential for maintaining the reduced state of glutathione. Together, these changes reflect a coordinated antioxidant response aimed at maintaining redox balance under elevated mitochondrial activity.

In contrast to the upregulation of oxidative metabolism, KEGG analysis identified protein processing in the endoplasmic reticulum (ER) as the most significantly downregulated pathway in HCD-fed mice (adj. P = 3.16 × 10^-14^; **Figure 3C**). This suppression was characterized by broad transcriptional downregulation of genes involved in ER function, protein folding and degradation, suggesting a metabolic shift away from proteostasis toward energy conservation (**Figures 5C, D**). Key molecular chaperones—including Hspa5, Hspa8, Hspa1a, Hspa1b, Hspa4l, Hsp90aa1, Hsp90ab1, Hsp90b1, Hsph1, Dnaja1, Dnajb1, Dnajb2, and Dnajb11—were significantly suppressed, indicating reduced protein folding capacity. Similarly, unfolded protein response (UPR) regulators such as Xbp1, Atf6, Ern1, Hyou1, and Map3k5 were downregulated, reflecting diminished ER stress signaling. Components of the ER-associated degradation (ERAD) pathway—including Syvn1, Sel1l, Derl3, Amfr, and Ubxn4—were also reduced, along with genes involved in vesicular trafficking (Sec24a, Sec24c, Sec24d, Sec23b, Sec31a, Sar1a, Sec61a1, Ssr3), suggesting a global decline in secretory activity. Additionally, genes responsible for oxidative protein folding and glycoprotein processing (Pdia4, Pdia6, Ero1l, Uggt1, Ostc, Stt3a, Ganab, Rpn1, Lman1, Tmem258, Ggt6, Wfs1) were consistently downregulated. Functionally, this coordinated suppression of ER activity may reduce energy expenditure associated with protein degradation, thereby preserving essential plasma proteins during systemic stress. Supporting this, plasma total protein and albumin levels were significantly higher in HCD-fed mice compared to RD controls, suggesting improved protein retention during sepsis. Finally, to assess functional relevance, we treated HCD-fed mice with rotenone, a mitochondrial complex I inhibitor, and evaluated survival following CLP-induced sepsis. Rotenone treatment significantly reduced survival compared to untreated controls, confirming that mitochondrial respiration is a critical mediator of high cholesterol diet-induced hepatic reprogramming and host defense (**Figure 3H**). To assess potential confounding effects of dietary intake, we monitored food consumption and body weight during the feeding period prior to CLP. Mice in the HCD group exhibited a modest decrease in food intake, but no significant differences in body weight were observed between HCD- and RD-fed mice (**Supplementary Figures 7B, C**). Given that the HCD contains 0.5% sodium cholate, we further evaluated whether bile acid–related signaling pathways were activated under our experimental conditions. Gene set enrichment analysis (GSEA) of liver RNA-seq data using all expressed genes and GO-defined bile acid signaling pathways showed no significant enrichment in HCD-fed mice compared with RD controls (NES = 0.676, FDR q = 0.841) (**Supplementary Figure 7D**).

**Figure 6:**
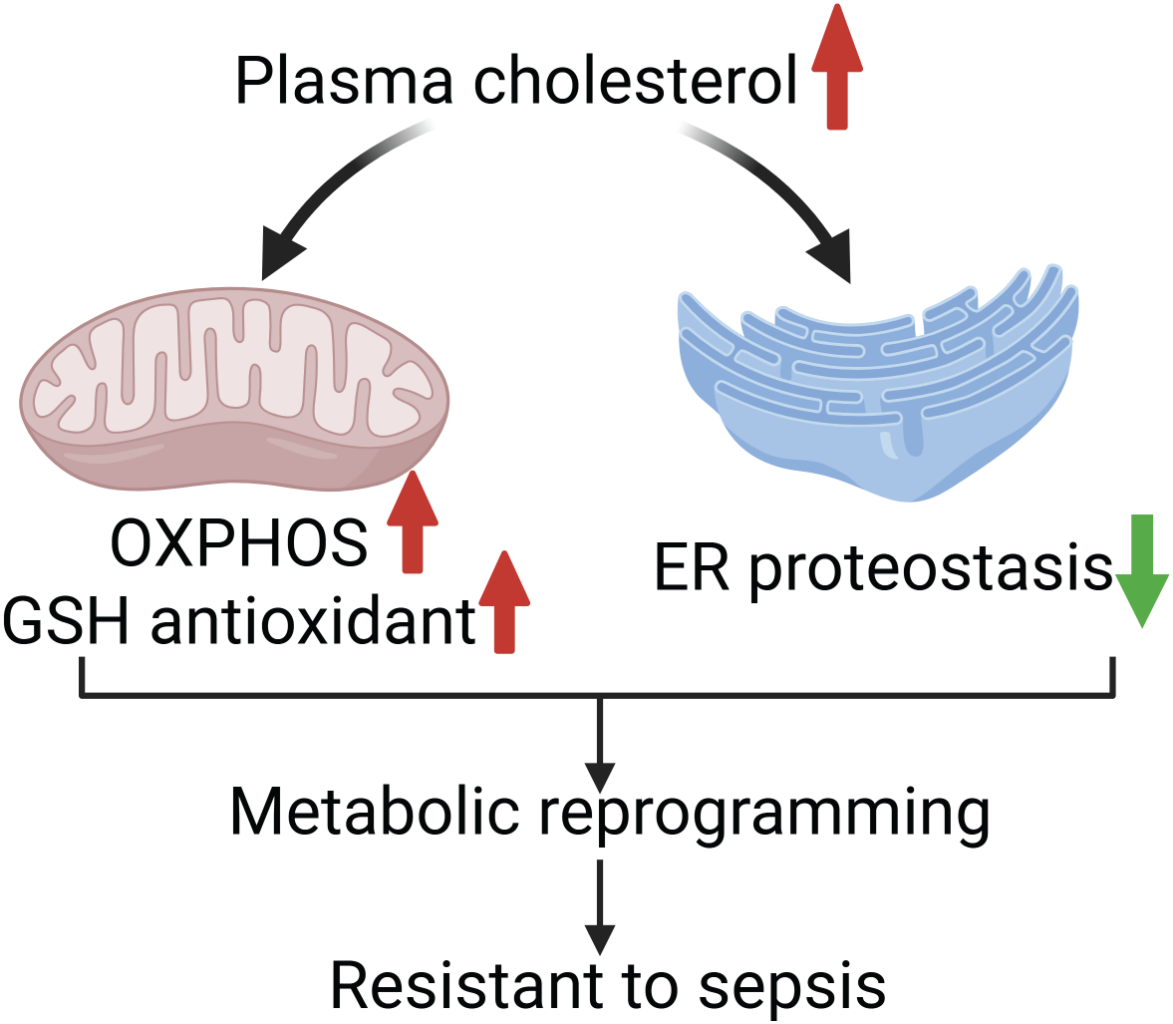
Schematic model of cholesterol-mediated protection in sepsis. Elevated plasma cholesterol confersa survival advantage in sepsis by promoting hepatic metabolic reprogramming. This includes upregulation of mitochondrial oxidative phosphorylation and glutathione-mediated antioxidant defenses, alongside attenuation of the energetic burden imposed by endoplasmic reticulum (ER) proteostasis. These coordinated adaptations enhance cellular energy balance and reduce oxidative stress, contributing to improved outcomes in sepsis. These protective effects highlight hepatic bioenergetics as a potentially targetable mechanism for sepsis therapy. Created with Biorender.com.

Collectively, these findings suggest that high-cholesterol diet drives a comprehensive hepatic metabolic reprogramming—characterized by activation of mitochondrial respiration and antioxidant defenses, alongside suppression of ER proteostasis. This coordinated shift prioritizes energy efficiency and redox balance, thereby enhancing systemic resilience to sepsis-induced stress (**Figure 6**).

## Discussion

The longstanding association between low plasma cholesterol and poor outcomes in sepsis has led to speculation about a protective role for cholesterol during systemic infection (9, 11). However, prior clinical studies were constrained by small sample sizes and insufficient adjustment for confounding variables (25–27). Using a large, well−curated cohort from the MIMIC−IV database and incorporating extensive adjustment for potential confounders, our study demonstrates that hypocholesterolemia is independently associated with increased mortality in critically ill patients with sepsis. Non−survivors exhibited significantly lower plasma total cholesterol than survivors, and patients in the low−cholesterol group had markedly worse 28−day ICU survival after adjustment for age, sex, race, SOFA score, and Charlson comorbidity index. Notably, this association persisted across multiple SOFA score strata, indicating that the relation.

Using a murine model of sepsis, we found that dietary cholesterol supplementation increased plasma cholesterol levels and markedly improved survival. Notably, this protective effect was not driven by broad changes in cytokine production, leukocyte recruitment, or bacterial clearance. Instead, the findings shift attention from immunomodulation toward metabolic adaptation as a key determinant of host resilience. Mechanistically, HCD feeding triggered a hepatic metabolic reprogramming marked by enhanced oxidative phosphorylation and antioxidant capacity, alongside suppression of endoplasmic reticulum (ER) proteostasis. Together, these coordinated metabolic adjustments optimize energy generation, limit oxidative stress, and reduce protein catabolism during sepsis. The functional importance of this metabolic shift was further demonstrated by pharmacological inhibition of mitochondrial respiration, which eliminated the survival advantage conferred by cholesterol supplementation, suggesting mitochondrial metabolism as a central mediator of host protection. In consistent with our study, an early report showed that enhancing OXPHOS improves outcomes in sepsis. In a murine sepsis model, dichloroacetate (DCA) inhibited pyruvate dehydrogenase kinase (PDK), thereby reactivating the pyruvate dehydrogenase complex and promoting OXPHOS. DCA treatment markedly improved survival, increasing survival rates from 19% to 71% (28). Together, the loss of protection following OXPHOS inhibition and the improved survival observed with OXPHOS enhancement support a model in which metabolic reprogramming toward increased OXPHOS contributes to host resilience and improved survival during sepsis.

Sepsis induces a metabolic shift from mitochondrial OXPHOS to glycolysis, particularly in immune and parenchymal cells. While glycolysis supports rapid ATP generation and acute immune activation, it is energetically inefficient and contributes to immunosuppression and organ dysfunction over time. Restoring mitochondrial OXPHOS has thus emerged as a promising therapeutic strategy. Our findings reinforce this concept. Although cholesterol was used as a pre-treatment in our study, the transcriptional programs identified—especially those enhancing mitochondrial function and antioxidant capacity—represent potential therapeutic targets. Modulating these pathways through pharmacological agents or tailored nutritional interventions may improve host resilience, even in the absence of prior cholesterol elevation. In addition to therapeutic implications, our findings may have relevance for clinical risk stratification. Low baseline cholesterol levels have been associated with increased susceptibility to sepsis and worse outcomes. For example, preoperative hypocholesterolemia has been identified as a significant risk factor for postoperative sepsis in patients undergoing urologic surgery (29). These observations support the concept that baseline cholesterol status may help identify individuals at higher risk for sepsis and its complications.

Of note, a few cytokines (IL−1β, KC, and MIP−2) showed a moderate decrease (2–3−fold) at 4 hours after CLP in high−cholesterol–fed mice, representing changes occurring during the early stage of sepsis. However, at 20 hours post−CLP, these cytokines did not differ significantly between diet groups (**Supplementary Figure 3E**). This pattern suggests that the transient early decrease may not translate into a biologically meaningful alteration of the overall inflammatory response. To further evaluate functional consequences, we assessed whether the modest decreases in KC and MIP−2 affected leukocyte recruitment to the infection site (**Supplementary Figures 4D, G**). Because KC and MIP−2 are potent neutrophil chemoattractants (30), we examined peritoneal leukocytes using flow cytometry. We found no significant decrease in neutrophil recruitment at 4, 20, or 44 hours post−CLP in high−cholesterol–fed mice, indicating a limited functional impact. Regarding IL−1β, this cytokine primarily acts upstream to induce secondary mediators such as IL−6. In our data, IL−6 levels did not differ between groups at 4, 20 or 44 post CLP (**Supplementary Table 2**), again suggesting that the early IL−1β decrease did not propagate into an altered downstream cytokine response. Taken together, although a few cytokines exhibited a moderate early decrease, the overall inflammatory response—both functionally and over time—may not significantly contribute to increased survival in high cholesterol-fed mice.

We observed a divergence between albumin (Alb) mRNA and protein levels. While Alb transcript levels were not significantly altered between HCD- and RD-fed mice, circulating albumin and total protein levels were increased in the HCD group. Consistent with this, our RNA-seq analysis revealed downregulation of pathways involved in protein processing and degradation, including components of endoplasmic reticulum (ER) proteostasis. Reduced protein degradation or altered protein processing may contribute to increased circulating albumin without corresponding changes in mRNA levels. These findings suggest that cholesterol-induced metabolic reprogramming may shift hepatic proteostasis toward protein preservation, potentially supporting systemic protein homeostasis during sepsis.

Notably, our findings contrast with several prior studies reporting that high-fat or high-cholesterol diets impair mitochondrial OXPHOS (15, 31–33). Several factors may explain this discrepancy. First, most previous studies employed long-term dietary interventions spanning several months, whereas our study utilized a short-term feeding protocol. Second, our HCD included cholate, a bile acid that markedly elevates plasma cholesterol levels and may amplify metabolic effects. Third, earlier studies relied on targeted approaches such as RT-PCR or Western blotting, which focus on a limited set of mitochondrial markers and may miss broader transcriptional changes. Moreover, RT-PCR normalization typically assumes stable expression of housekeeping genes, yet our RNA-seq data revealed that β-actin was significantly upregulated (1.5-fold) in HCD-fed mice compared to RD controls. In contrast, our use of unbiased RNA sequencing enabled comprehensive profiling of transcriptional changes, revealing systemic metabolic reprogramming—including robust upregulation of OXPHOS genes across all mitochondrial complexes and antioxidative pathways, alongside suppression of ER proteostasis pathways—that targeted methods may overlook.

Our study has several limitations. First, the observational nature of the clinical data precludes causal inference, and residual confounding cannot be fully excluded despite multivariable adjustment for key demographic factors, comorbidities, and disease severity indices. The use of cholesterol-lowering medication prior to ICU admission was unknown and thus not considered in the analysis. Another limitation is the variability in the timing of total cholesterol measurements. Although we prioritized values obtained within the first 24 hours of ICU admission, some measurements were taken from the earliest available laboratory results during the same hospitalization when ICU−day values were not available. In certain cases, these measurements may have been obtained prior to ICU admission or influenced by early clinical management. This variability is inherent to retrospective analyses using electronic health record data and may introduce potential bias in estimating baseline cholesterol levels. Second, the murine model may not fully recapitulate the complexity and heterogeneity of human sepsis, particularly with respect to comorbidities, microbiota, and immune responses. Murine sepsis models produce relatively synchronized inflammatory and metabolic responses, while human sepsis evolves over variable and often prolonged time courses that include overlapping hyperinflammatory and immunosuppressive phases (34, 35). Nevertheless, rodent models remain indispensable for dissecting causal mechanisms that cannot be readily addressed in humans, particularly for isolating the effects of specific metabolic perturbations under controlled conditions. As emphasized in recent translational frameworks, the strength of murine sepsis models lies not in reproducing the full clinical complexity of human sepsis, but in enabling mechanistic discovery that can be subsequently tested and refined in human cohorts. In this context, our integration of large-scale clinical data with mechanistic mouse studies provides complementary evidence supporting a role for cholesterol-driven hepatic metabolic reprogramming in sepsis resilience, while highlighting the need for future validation in human tissues and interventional studies. Thirdly, while rotenone is widely used as a mitochondrial complex I inhibitor, it has been reported to induce microglial activation and promote astrocyte–microglia crosstalk (36). Similarly, *in vitro* studies demonstrate that rotenone can trigger inflammatory responses, particularly in microglial and neuronal cell systems (37). These findings suggest the possibility that systemic rotenone exposure may also influence immune cell function in our model. Thus, these findings should be interpreted as supportive, but not liver-specific, evidence for the role of mitochondrial function in sepsis resilience. Additional studies are also needed to validate transcriptomic findings at the functional levels, such as ATP measurements or oxygen consumption analyses. While transcriptomic data support activation of glutathione-related pathways, direct biochemical validation would provide further insight into antioxidant function.

### Translational implications and challenges

Translating this finding into clinical practice presents both opportunities and significant challenges. 1) Observational data suggest that hypocholesterolemia is a marker of poor prognosis in sepsis and high-risk surgical patients (29). Both hypocholesterolemia and hypercholesterolemia can be harmful, and the optimal level may depend on patient-specific factors such as comorbidities, genetic background, and phase of illness. However, indiscriminate cholesterol supplementation could pose risks related to cardiovascular disease, immune modulation, or lipid overload, especially in patients with pre-existing metabolic disorders. Therefore, careful patient stratification—potentially targeting those with marked hypocholesterolemia or impaired lipid transport during early sepsis—will be essential. 2) Our murine model demonstrates that short-term elevation of plasma cholesterol prior to septic insult promotes protective hepatic reprogramming. In human sepsis, the therapeutic window is likely narrow. Interventions initiated too late, after organ failure and immunosuppression are established, may fail to restore metabolic resilience. This underscores the need for early, possibly even prophylactic, intervention in high-risk patients. 3) Although researchers have explored cholesterol supplement approaches, such as administering fish oil-containing lipid emulsions or developing nanoparticle-based delivery systems, these approaches remain primarily investigational and lack definitive clinical validation in sepsis (7). Therefore, more direct and readily translatable interventions—including tailored dietary regimens in high-risk populations or pharmacological agents that directly enhance mitochondrial oxidative phosphorylation warrant further investigation. 4) While acute cholesterol elevation improved survival in our study, persistent alterations in lipid metabolism post-sepsis could influence long-term cardiovascular risk, hepatic function, and immune homeostasis. Future translational studies must incorporate longitudinal follow-up to assess these potential sequelae. Thus, future research should integrate patient phenotyping, targeted metabolic interventions, and carefully designed early-phase trials to determine if modulating these pathways can improve outcomes in sepsis.

Importantly, in our study, the HCD was administered prior to CLP, modeling a condition in which individuals enter sepsis with elevated baseline cholesterol levels, rather than representing a therapeutic intervention after sepsis onset. Dietary cholesterol increases plasma cholesterol levels too slowly to serve as an effective intervention during the acute phase of sepsis, when rapid metabolic adaptation is required. Together, these findings indicate that cholesterol-mediated protection is more likely related to baseline metabolic preparedness rather than acute therapeutic supplementation. These observations have important clinical implications for risk stratification, suggesting that baseline cholesterol levels may serve as a biomarker to identify individuals at increased risk for sepsis and poor outcomes, and may inform early preventive or metabolic optimization strategies in high-risk populations.

In summary, our study suggests cholesterol as a critical regulator of host resilience during sepsis. By promoting hepatic metabolic reprogramming—enhancing mitochondrial respiration and antioxidant defenses, while alleviating the energetic burden of ER proteostasis—cholesterol supplementation may confer a survival advantage that appears independent of classical immune pathways. These findings offer potential mechanistic insight into longstanding clinical observations linking hypocholesterolemia with poor sepsis outcomes and suggest that targeting hepatic metabolism may represent a novel therapeutic strategy. Future studies should explore pharmacological approaches that mimic the beneficial metabolic programs induced by HCD, potentially offering new avenues to improve outcomes in sepsis and related critical illnesses.

## Data Availability

The RNA-seq data generated in this study have been deposited in the Gene Expression Omnibus (GEO) under accession number GSE329833, https://www.ncbi.nlm.nih.gov/geo/query/acc.cgi?acc=GSE329833.
